# Mendelian randomization studies of risk and protective factors for osteoporosis: a systematic review and meta-analysis

**DOI:** 10.3389/fendo.2024.1486188

**Published:** 2025-01-16

**Authors:** Wenhao Ji, Bin Pan, Xin Chen, Zhaobai Lao, Wanlei Yang, Yu Qian

**Affiliations:** ^1^ Department of Orthopedics Surgery, The First Affiliated Hospital of Zhejiang Chinese Medical University (Zhejiang Provincial Hospital of Chinese Medicine), Hangzhou, Zhejiang, China; ^2^ The First Clinical Medical College, Zhejiang Chinese Medical University, Hangzhou, Zhejiang, China

**Keywords:** osteoporosis, Mendelian randomization, risk factors, genetic epidemiology, meta-analysis

## Abstract

**Background:**

Mendelian randomization is believed to attenuate the biases inherent in observational studies, yet a meta-analysis of Mendelian randomization studies in osteoporosis has not been conducted thus far. This study aims to evaluate the connection between potential causal factors and the risk of osteoporosis by synthesizing evidence from Mendelian randomization studies.

**Methods:**

The databases PubMed, Web of Science, and Embase were systematically searched for Mendelian randomization studies investigating factors influencing osteoporosis up to May 2024. Meta-analyses were conducted to assess the associations between various potential pathogenic factors and osteoporosis using Mendelian Randomization studies. The quality of the study was evaluated according to the Strengthening the Reporting of Observational Studies in Epidemiology via Mendelian Randomization (STROBE-MR) guidelines.

**Results:**

A total of 706 potentially relevant articles were screened, resulting in the inclusion of 53 studies in the systematic review, of which 30 were eligible for the meta-analysis. The combined findings from these 30 studies revealed that rheumatoid arthritis, inflammatory bowel disease, sex hormone binding globulin, depression, non-alcoholic fatty liver disease, primary biliary cholangitis and asthma are associated with increased risk of osteoporosis, while basal metabolic rate and gut microbiota (NB1n) serves as a protective factor. However, the association between obesity, type 2 diabetes mellitus, metformin, ulcerative colitis, leisure sedentary behaviors, systemic lupus erythematosus, serum iron and osteoporosis was found to be nonsignificant.

**Conclusion:**

In summary, our meta-analysis indicates that significant causal relationships with osteoporosis’s onset and progression have been established for rheumatoid arthritis, inflammatory bowel disease, primary biliary cholangitis, non-alcoholic fatty liver disease, depression, sex hormone binding globulin, basal metabolic rate, gut microbiota (NB1n), and asthma.

**Systematic review registration:**

https://www.crd.york.ac.uk/prospero/, identifier PROSPERO CRD42024540504.

## 1 Introduction

Osteoporosis (OP) represents a systemic skeletal disorder marked by diminished bone density, compromised bone architecture, heightened susceptibility to fractures, and increased brittleness (1). With the aggravation of the global aging problem, the incidence of OP is positively correlated with age, which has become an important public health problem facing the world today (2). Fractures are among the gravest complications arising from OP (3). Epidemiological data indicates that close to 9 million fractures attributable to OP transpire annually across the globe, culminating in diminished life quality and elevated mortality risk (4).

Numerous epidemiological investigations have identified potential determinants of OP, such as low physical activity, insufficient sunlight exposure, smoking, excessive alcohol consumption, nutritional imbalances and so on (5). However, most studies investigating the risk factors of OP are mainly based on observational study design. Moreover, observational studies are susceptible to the interference of reverse causality and confounding factors, thus limiting the inference of causality. Although randomized controlled trials (RCTs) are the “gold standard” for examining causality, RCTS require a significant investment of time and money, and are often not suitable for all research questions due to ethical and financial constraints. Mendelian randomization (MR) offers a viable alternative for exploring causal relationships, utilizing genetic variants as instrumental variables to assess the causal impact of various exposures on health outcomes. Its unique methodological features and advantages of less confusion bias interference make it a powerful tool to evaluate the relationship between exposure factors and disease onset (6). Many MR studies have investigated the causal relationship between lifestyle, nutrition, disease status, and OP. However, these studies have differences in research design, population composition, genetic tools, and methods for estimating causal effects, which may lead to biased or uncertain results.

Numerous systematic reviews have been performed on MR studies, encompassing various conditions such as cancer (7), arthritis (8), and rheumatoid arthritis (RA) (9). To the best of our knowledge, the present study is the inaugural to evaluate and summarize existing MR research through a systematic review and meta-analysis, with the aim of identifying potential risk and protective factors associated with OP.

## 2 Materials and methods

### 2.1 Data sources and search strategy

**Figure 1:**
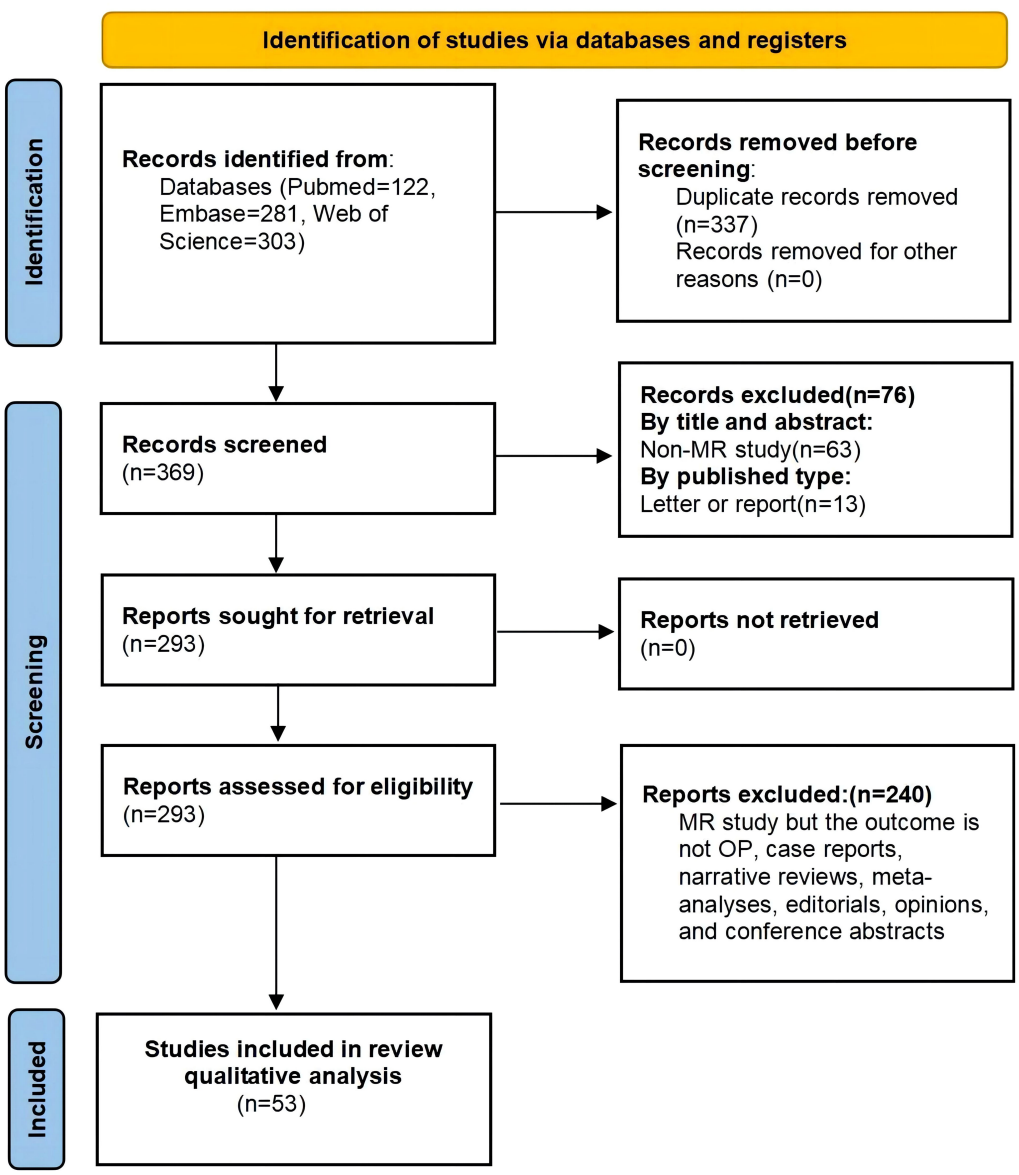
Preferred Reporting Items of Systematic Review and Meta-analyses (PRISMA) flow diagram.

This review was meticulously carried out, adhering to the PRISMA-P 2020 guidelines (10, 11). It has been officially registered in the PROSPERO database (CRD42024540504). The process illustrated in **Figure 1** outlines the procedures for literature retrieval and inclusion-exclusion. PubMed, Embase, and Web of Science electronic databases were searched for relevant MR literature on OP up to May 1, 2024. The complete search strategy for each database is outlined in **Supplementary Table S1**.

### 2.2 Study selection

The articles retrieved were imported into the NoteExpress reference library, version 3.9 (Beijing Aegean Lezhi Technology, China), for the identification and removal of duplicates. Subsequently, two reviewers independently assessed the remaining articles, selecting only those meeting the predetermined criteria. In cases of disagreement, a third investigator was consulted to achieve a consensus.

Study selection criteria:

Inclusion:

1. Original studies investigating causal relationships between risk factor-associated phenotypes and OP using MR analysis.
2. Any studies that include MR as part of their analysis.
3. Studies encompassing diverse sex, age, cohorts, and ethnicities.

Exclusion:

1. Any case reports, narrative reviews, or other non-research-based studies.
2. Studies lacking complete manuscripts or original datasets.
3. Research that does not consider OP as an outcome.

### 2.3 Data extraction and quality assessment

We extracted data from each MR study, including the first author’s surname, year of publication, alliances or consortia involved in OP genomics research, total participant count, the ancestry of the cohort, investigated exposure factors, and principal outcomes. Quality assessment followed the Strengthening the Reporting of Mendelian Randomization Studies (STROBE-MR) Guidelines (12). After converting the quality assessment score to a percentage, studies were categorized as high-quality (scores > 85%), medium-quality (scores between 75% and 85%), and low-quality (scores < 75%) accordingly (**Supplementary Table S2**).

### 2.4 Statistical analysis

When a minimum of two separate studies or datasets were available to evaluate the causality between pathogenic factors or genetic markers and OP, the gathered data were assimilated for meta-analytic evaluation. To determine the combined OR values for various factors influencing OP risk, both fixed-effect and random-effects models were applied. Study heterogeneity was gauged using Cochran’s Q test and Higgins’ I² test. If the summary results of the fixed effects model are obtained with I² > 50% and p < 0.05, it is considered that the heterogeneity is statistically significant, and the results of the random effects model should be reported. All data analyses were performed using RevMan version 5.4.1 (Copenhagen: The Nordic Cochrane Centre, The Cochrane Collaboration, 2020), with statistical significance established at a two-sided p-value below 0.05.

## 3 Results

### 3.1 Study selection

The initially retrieved database yielded 706 records using the pre-established retrieval strategy. However, upon closer inspection, only 53 articles were ultimately included in this review, as illustrated in **Figure 1**.

### 3.2 Study characteristics

The systematic review encompassed a total of 53 MR studies (13–65). These studies included participants with OP from various datasets, representing both European and East Asian populations. The studies utilized a wide range of single nucleotide polymorphisms (SNPs), from a minimum of 3 (53) to a maximum of 462 (33). All MR studies utilized data from multiple sources, including the IEU OpenGWAS Project, GEnetic Factors for OSteoporosis Consortium (GEFOS), UK Biobank, FinnGen biobanks, Biobank Japan and The Neale Lab. **Table 1** provides a detailed overview of the characteristics of the included studies. All studies provided comprehensive information on sample sizes for both exposure and outcome variables. While two study did not specify the number of SNPs used in the MR analysis (62, 63), the majority of studies employed a range of 3 to hundreds of SNPs as instrumental variables. In terms of selecting SNPs, most MR studies adopted a stringent threshold for linkage disequilibrium (R² < 0.001) to ensure the in-dependence of the SNPs used as instrumental variables for exposure. Additionally, all studies clearly delineated the statistical analysis methods employed for MR analysis. 22 studies presented sensitivity analysis results using robust MR methods such as Inverse Variance Weighting (IVW), and assessed horizontal pleiotropy using the MR-Egger method (16–18, 22, 23, 25, 27–29, 31, 33, 38–43, 46–49, 54, 58, 65). This transparency in methods and rigorous approach to data analysis enhance the overall reliability and validity of the findings presented in these studies (**Table 1**).

**Table 1:** Characteristics of 53 studies included in qualitative analysis.

Study	Year	Ethnicity	Cohort	Exposure	Sample size	Findings
Wu et al. (13)	2024	Asian	UKB	Periodontitis	7,788cases, 20,4665conttrols	No causality between periodontitis and OP.
Lee et al. (14)	2024	European	UKB, FinnGen	Predicted plasma cortisol	9,734cases, 58,0320controis	Non-causal association between plasma cortisol and OP.
Zhang et al. (15)	2024	European	IEU	H. pylori infection	5,266cases, 33,1893controls	H. pylori infection causally associated with OP risk.
Sun et al. (16)	2023	European	FinnGen	LTL/SHBG	7,300cases, 35,8014conrols	Longer LTL and the levels of SHBG causally associated with OP risk.
Chen et al. (17)	2024	European	IEU	Leisure sedentary behaviors	7,547cases, 45,5386controls	No causality between leisure sedentary behaviors and OP.
Chen et al. (18)	2024	European	FinnGen	Asthma	7,300cases, 35,8014conrols	Asthma causally associated with OP risk
Li et al. (19)	2024	European	FinnGen	Cortical structure	7,300cases, 35,8014conrols	Cortical structure causally associated with OP risk
Dou et al. (20)	2024	European	UKB	COPD	5,266cases, 33,1893controls	COPD causally associated with OP risk.
Zhang et al. (21)	2024	European	FinnGen	Daytime napping	30,2610	Daytime napping causally associated with OP risk.
Wu et al. (22)	2024	European, Asian	UKB, FinnGen, BBJ	Primary biliary Cholangitis (PBC)	6,484cases,40,1279controls/6,303cases, 32,5717controls/ 9,794cases, 16,8932controls	PBC causally associated with OP risk.
Shi et al. (23)	2024	European, Asian	UKB, BBJ	Systemic lupus erythematosus (SLE)	7,547cases,45,5386controls/7,788cases, 20,4665controls	In the East Asian population, SLE causally associated with OP risk; In Europe, no causality between SLE and OP.
Huang et al. (24)	2024	European	FinnGen	SHBG	21,2778	Circulating SHBG levels causally associated with OP risk.
Ding et al. (25)	2024	European	FinnGen	COA and AOA	3,203cases, 20,9575controls	Both COA and AOA have a genetically causal effect on OP.
Li et al (26)	2024	European	FinnGen	Neurodevelopmental disorders	36,5314	No causality between neurodevelopmental disorders and OP.
Liu et al. (27)	2024	European	FinnGen	NAFLD/ PBC	6,303cases, 32,5717controls	PBC causally associated with OP risk.
Zeng et al. (28)	2024	European	UKB	Gut microbiota (GM)	5,266cases, 33,1893controls	GM causally associated with OP risk.
Cai et al (29)	2024	European	FinnGen	Metformin	3,204cases, 20,9575controls	Metformin causally associated with OP risk.
Guo et al. (30)	2024	European	EBI	Major depressive disorder (MDD)	48,4598	MDD causally associated with OP risk.
Wu et al. (31)	2024	European	FinnGen	Rheumatoid arthritis (RA)	21,2778	RA causally associated with OP risk.
Duan et al. (32)	2024	European	FinnGen	Socio-economic status	36,5314	Socio-economic status causally associated with OP risk.
Zhou et al. (33)	2023	European	IEU, Neale Lab	Basal metabolic rate	7,547 cases, 45,5386 controls/5,266 cases, 33,1893 controls	Basal metabolic rate causally associated with OP.
Li et al. (34)	2023	European	UKB	Diet-derived antioxidants	7,547cases, 45,5386controls	Serum β-carotene could elevate BMD and prevent osteoporosis.
Li et al. (35)	2023	European	IEU	Selenium Levels	7,751cases, 47,6847controls	No causality between Se levels and OP risk.
Zhang et al. (36)	2023	European	UKB	COVID-19	7,547cases, 455,386controls	No causality between the severity of COVID-19 and OP risk.
Tang et al. (37)	2023	European	FinnGen, UKB	IGF family members	3,203cases,20,9575controls/5,266cases, 33,1893controls	IGF family members causally associated with OP risk
Cui et al. (38)	2023	European	UKB	NAFLD	7,547 cases and 455,386 controls	A causal association between NAFLD and osteoporosis.
Dai et al. (39)	2023	European	IEU	Inflammatory bowel disease	3,203cases, 20,9575controls	CD causally associated with OP risk.
Wei et al. (40)	2023	European	FinnGen	Metformin	7,300cases, 358,014controls	Metformin use causally associated with OP.
Huang et al. (41)	2023	East Asian population	BBJ	Type 2 diabetes mellitus	7,788cases, 20,4665conttrols	T2DM is not associated with reduction in BMD.
Zhao et al. (42)	2023	European	FinnGen	PBC	7,300cases, 358,014controls	PBC causally associated with OP risk.
Zhou et al. (43)	2023	European	IEU	NAFLD	7547 cases, 455,386 controls	NAFLD causally associated with OP.
Tang et al. (44)	2023	European	FinnGen	Type 1 diabetes	5,354cases, 294,793controls	No causality between T1D and OP risk.
Du et al. (45)	2023	European	FinnGen	Vitamin D/ sex hormones	6,303 cases, 325,717 controls	25(OH)D and TT had a causal effect on osteoporosis.
Cheng et al. (46)	2023	European	UKB	Type 2 diabetes mellitus	7,547 cases, 455,386 controls	A causal link between DM2 and OP.
Tang et al. (47)	2023	European	FinnGen	Mental diseases	3,203cases, 209,575controls	No causality between MDs and OP risk.
Deng et al. (48)	2023	Japanese	BBJ	RA	7,788cases, 20,4665conttrols	RA causally associated with OP risk.
Xu et al. (49)	2023	East Asian	BBJ	Inflammatory bowel disease	7,788cases, 20,4665conttrols	Inflammatory bowel disease causally associated with OP risk.
Sun et al. (50)	2023	European	FinnGen	Educational attainment	6,303cases, 325,717controls	No causality between EA and OP.
Lyu et al. (51)	2023	European	IEU	Zinc, immunity, physical activity	33,7159	Zinc causally associated with OP risk
Chen et al. (52)	2023	European	IEU	Lipid- lowering therapies	7,547 cases, 455,386 controls	Lipid-lowering variants of PCKS9 and HMGCR were associated with decreased risks of OP.
Gagnon et al. (53)	2023	European	IEU	GM and associated metabolites	7,547 cases, 455,386 controls	No causality between gut microbita features and OP risk.
Chen et al. (54)	2023	European	UKB	Specific GM	7,547 cases and 455,386 controls	Specific bacteria taxa causally associated with OP risk
Ji et al. (55)	2023	European	IEU	Cytokines	7,547cases, 45,5386controls	Cytokines causally associated with OP risk
Zahn et al. (56)	2023	European	The Neale Lab	Targeting Longevity Gene *SLC13A5*	36,1194	SNPs linked to reduced *SLC13A5* function lowered osteoporosis risk
Xu et al. (57)	2022	European	UKB	Dietary Habits	9,434cases, 44,4941controis	Five dietary habits causally associated with OP
Xu et al. (58)	2022	European	FinnGen	Iron status	30,0147	No causality between iron status and OP.
Yuan et al. (59)	2022	European	UKB	Cystatin C	5,266cases, 33,1893controls	Serum cystatin C levels causally associated with OP
Chen et al. (60)	2022	European	FinnGen	Tea consumption	1,175cases, 93,083controls	No causality between tea consumption and OP.
Kasher et al. (61)	2022	European	UKB	RA	7,734 cases, 35,3 407controls	OP phenotypes did not show causal associations with RA
Martin et al. (62)	2022	European	FinnGen,IEU, UKB	BMI/BFP /FA/UFA	42,1084cases, 737,530controls/ 2,452cases, 16,9858controls/ 1,4663cases, 43,6361controls	Body fat percentage causally associated with OP risk.
Yu et al. (63)	2021	Asian	BBJ	RA	7,788cases, 21,2453controls	RA causally associated with OP risk.
Kou et al. (64)	2020	European	UKB	IL-18	933 cases, 360,261 controls	IL-18 level causally associated with OP risk.
Cheng et al. (65)	2019	European	GEFOS	Blood minerals	50,8253cases, 5,3236controls	Magnesium causally associated with OP risk.

OP, osteoporosis; UKB, UK Biobank; BBJ, BioBank Japan; IEU, IEU OpenGWAS project; EBI, European Bioinformatics Institute; GEFOS, GEnetic Factors for OSteoporosis Consortium; LTL, leukocyte telomere length; SHBG, sex hormone-binding globulin; NAFLD, Non-alcoholic fatty liver disease; T2DM, Type 2 diabetes mellitus; MDs, mental diseases; CD, Crohn’s disease; COA, childhood-onset asthma; AOA, adult-onset asthma; BFP, body fat percentage; BMD, bone minerali density; FA, favorable adiposity; UFA, unfavorable adiposity.

### 3.3 Meta-analysis results

A total of one studies (three datasets) for obesity (62), the four indicators are body mass index (BMI), body fat percentage (BFP), favorable obesity (FA) and unfavorable obesity (UFA), four studies for RA (31, 48, 61, 63), two studies for Type 2 diabetes mellitus (T2DM) (41, 46), two studies for inflammatory bowel disease (IBD) (39, 49), two studies for ulcerative colitis (UC) (39, 49), two studies for Crohn’s disease (CD) (39, 49), three studies for Non-alcoholic fatty liver disease (NAFLD) (27, 38, 43), three studies for primary biliary cholangitis(PBC) (22, 27, 42), one study (two datasets) for systemic lupus erythematosus (SLE), two studies for depression (30, 47), two studies for metformin (29, 40), one study (three datasets) for leisure sedentary behaviors (17), two studies for asthma (18, 25), three studies for gut microbiota (NB1n) (GM) (28, 53, 54), two studies for serum iron (58, 65), two studies for sex hormone binding globulin (SHBG) (16, 24), and one study (two datasets) for basal metabolic rate (BMR) were selected for quantitative analysis based on the presence of common risk factors across the studies for meta-analysis (33).

#### 3.3.1 Risk factors

**Figure 2:**
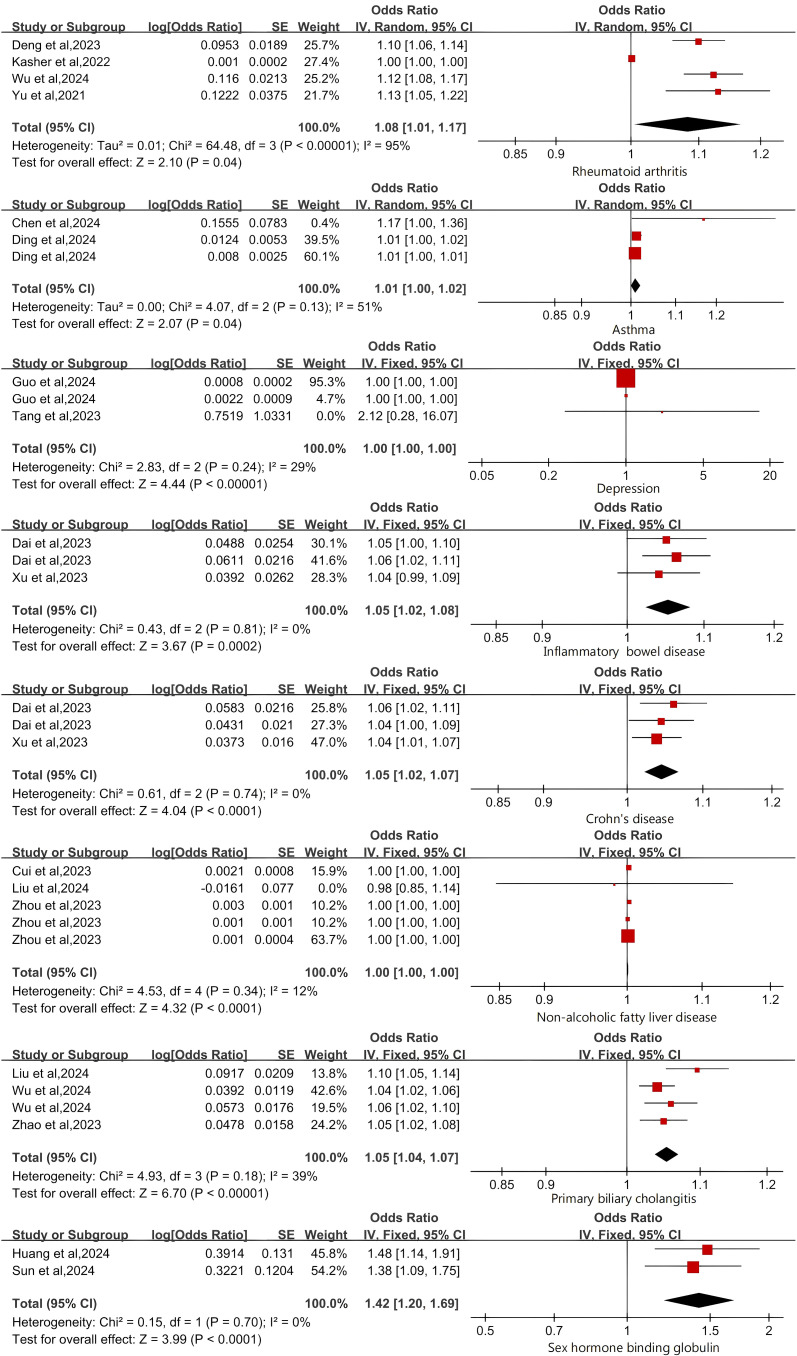
Forest Plot of Risk Factors for Osteoporosis.

We conducted a meta-analysis of the included MR studies. The results indicate that the presence of IBD may increase the risk of OP by 5% (OR 1.05 [1.02-1.08]). The presence of NAFLD may increase the risk of OP by 0.14% (OR 1.00 [1.00-1.00]). The presence of CD may increase the risk of OP by 5% (OR 1.05 [1.02-1.07]). The presence of RA may increase the risk of OP by 8% (OR 1.08 [1.01-1.17]). The presence of PBC may increase the risk of OP by 5% (OR 1.05 [1.04-1.07]). The presence of asthma may increase the risk of OP by 1% (OR 1.01 [1.00-1.01]). The presence of depression may increase the risk of OP by 0.09% (OR 1.00 [1.00-1.00]). SHBG is also a risk factor for OP (OR 1.42 [1.20-1.69]). The Higgins I² test identified significant heterogeneity in RA and asthma. Despite this, the random-effects model was selected owing to the small number of studies. In contrast, analyses without heterogeneity were conducted using the fixed-effects model as the main approach (**Figure 2**; **Supplementary Figure S1**).

#### 3.3.2 Protective factors

**Figure 3:**
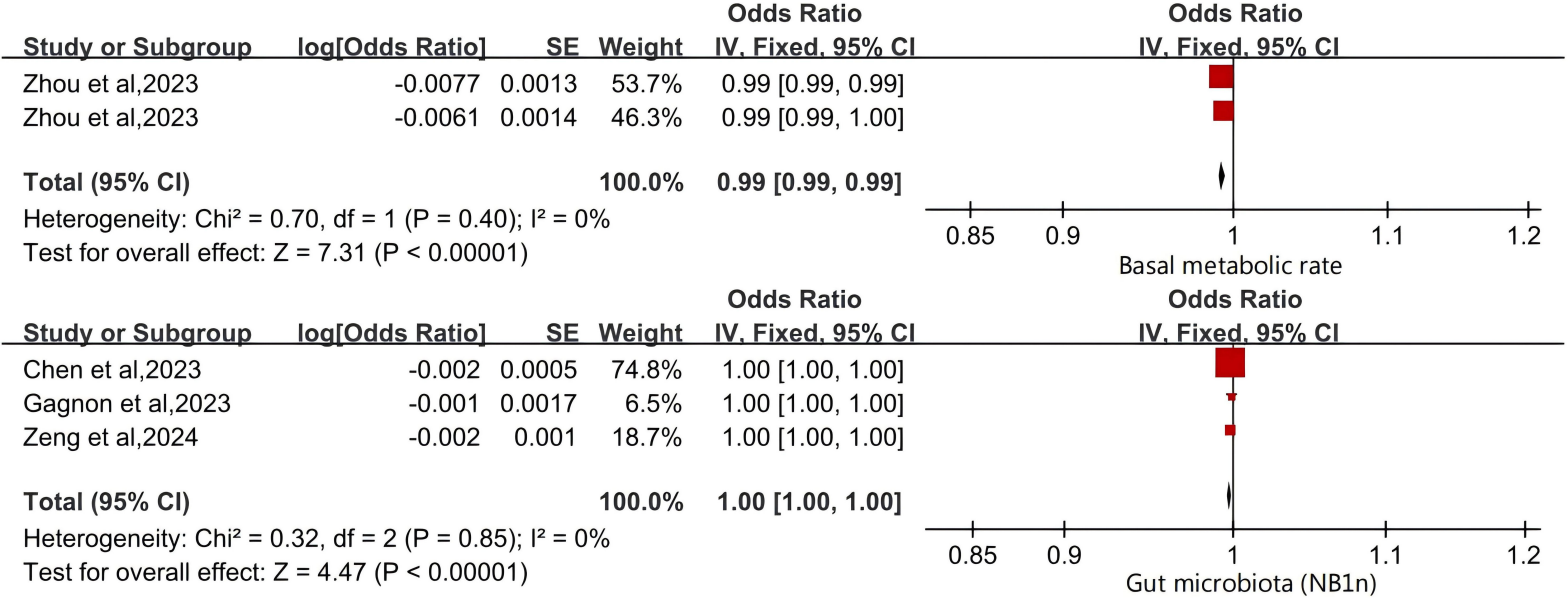
Forest Plot of Protective Factors for Osteoporosis.

We evaluated the overall causal effects of BMR and GM (NB1n) on OP using a fixed effects model. Our analysis results indicate that BMR (OR: 0.99 [0.99-0.99]) and gut microbiota (NB1n) (OR: 1.00 [1.00-1.00]) have a protective effect on OP outcomes (**Figure 3**). In addition, we further validated the results of this study using a random effects model (**Supplementary Figure S2**).

#### 3.3.3 Factors with no significant correlation

The genetic susceptibility of the four indicators related to obesity is not associated with an increased risk of osteoporosis. Cochran’s Q test and Higgins’ I ² test revealed significant heterogeneity among BMI, BFP, and UA studies. However, due to the limited number of studies included, we chose the random effects model for evaluation. There was no significant correlation between BMI, BFP, FA, UA, and OP predicted by genes (BMI (p=0.59), BFP (p=0.90), FA (p=0.26), UFA (p=0.75)).

**Figure 4:**
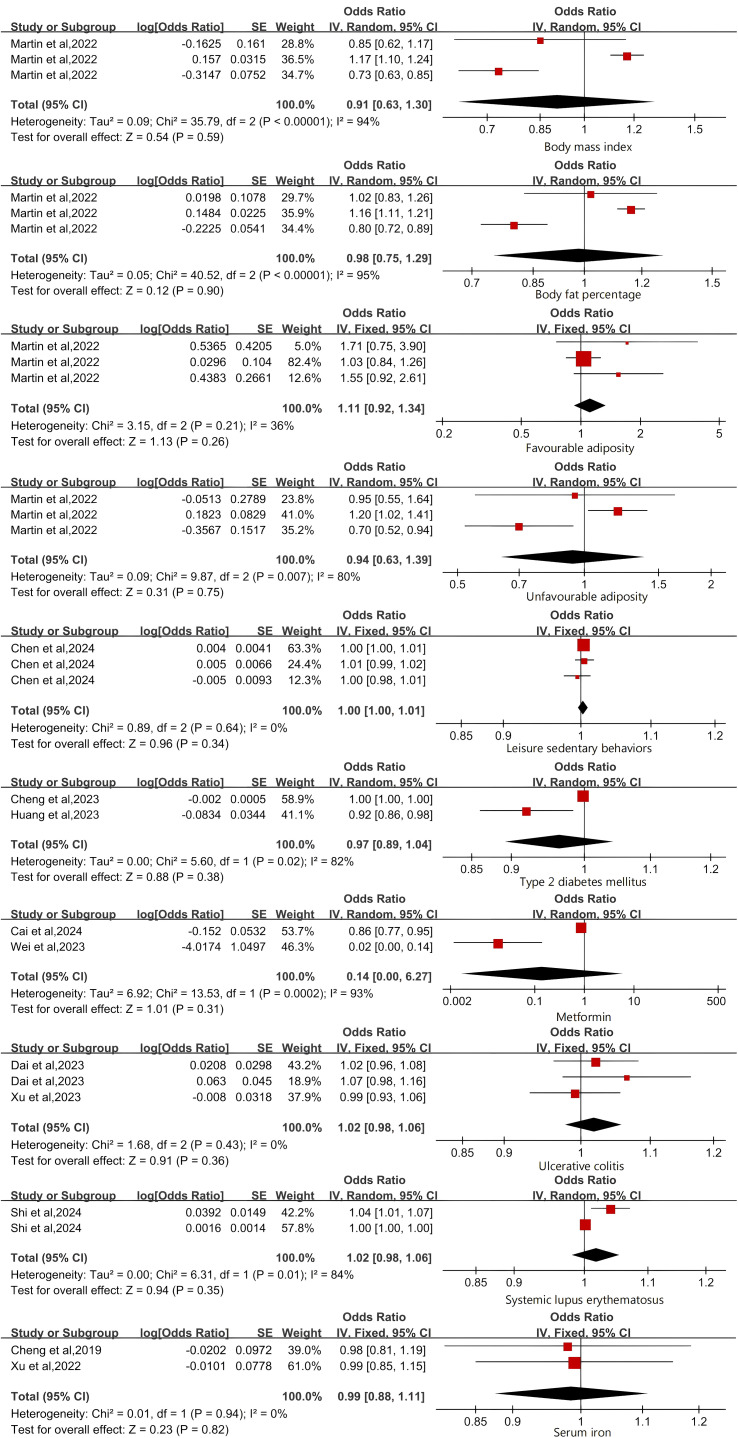
Forest Plot of Factors with No Significant Association with Osteoporosis.

T2DM (p = 0.38), metformin (p = 0.31), UC (p = 0.36), SLE (p = 0.35), leisure sedentary behaviors (p = 0.34) and serum iron (p=0.82) were not significantly associated with an increased risk of OP (**Figure 4**). We further validated the above results using a random effects model (**Supplementary Figure S3**).

## 4 Discussion

This analysis synthesizes MR findings from published literature. Genetic evidence shows that RA, IBD, PBC, NAFLD, SLE, asthma and SHBG correlate with a heightened risk of OP. Conversely, T2DM, metformin usage, BMR, and GM (NB1n) are linked to a reduced risk of OP. On the other hand, no association was found between obesity, sedentary leisure activities, serum iron levels, or depression and the risk of OP. These findings diverge somewhat from the quantitative analysis conducted on the 30 articles included in this review. Notably, the studies considered were of substantial quality and demonstrated minimal bias.

### 4.1 Risk factors

Meta-analysis found that RA predicted by genetics was positively correlated with OP. The findings align with the conclusions drawn from the articles incorporated in our systematic review. RA may lead to OP through a complex interaction between chronic inflammation and bone formation, osteolysis and resorption (66). This leads to the activation of the nuclear factor kappa B ligand receptor activator (RANKL)/nuclear factor kappa B receptor activator (RANK)/osteoprotegerin (OPG) signaling pathway, which in turn stimulates osteoclast differentiation while inhibiting osteoblast function, disrupting bone homeostasis, accelerating bone loss, and ultimately leading to OP (67–69). In addition, RA patients with long-term use of glucocorticoids have a higher risk of developing glucocorticoid-induced OP (70).

Asthma, a widespread chronic noninfectious condition, has been linked to an increased OP risk through clinical research (71). While past studies have largely focused on the prolonged use of glucocorticoids as a connecting factor between asthma and OP, the direct impact of asthma on bone health has not been fully considered (72). The research by Jee Youn Oh et al. indicates that comorbidities like asthma-COPD overlap can intensify the risk of OP, even when glucocorticoid effects are taken into account (73). Recent studies indicate that asthma may disrupt the interaction between osteoblasts and osteoclasts through inflammatory factors, accelerating bone turnover and leading to a disparity in bone resorption and formation, which may culminate in OP (18). Our meta-analysis also suggests a positive causal relationship between asthma and OP.

Depression is a mood disorder with symptoms that may vary from person to person, often leading to psychological and physical distress (30). A multitude of research efforts have sought to understand the connection between depression and OP. These studies suggest that depression could be a considerable risk factor for reduced BMD and fractures, despite some inconsistencies in findings (74). Our meta-analysis and included MR studies all show that depression may increase the risk of developing osteoporosis.

IBD, encompassing CD and UC, are idiopathic conditions thought to emerge from a blend of genetic predisposition and environmental factors (75). There has been an uptick in OP incidence and related pathological fractures among IBD sufferers, which may be because IBD patients often suffer from insufficient intake and malabsorption of nutrients due to chronic diarrhea and other factors, inflammatory factors such as interleukin (IL) promote osteoclast differentiation, and long-term use of glucocorticoid and other drugs lead to the imbalance of osteoblasts and osteoclasts, increasing the risk of OP (76–78). A population-based matched cohort study revealed a 40% higher fracture incidence in IBD patients compared to those without IBD (79). However, MR analysis has not been significant causal link between UC and OP. More extensive research is necessary to validate the association between IBD and OP.

NAFLD and PBC are common chronic liver diseases. Numerous studies have explored its potential link to OP, with findings indicating a higher prevalence of OP in NAFLD and PBC patients compared to those without the condition (80). The mechanisms through which chronic liver diseases may impact OP include alterations in bone metabolism, vitamin D status, chronic liver inflammation, hepatic fibrosis severity, and disturbances in lipid metabolism. Nonetheless, observational studies examining the link between chronic liver diseases and OP have produced varied results. While some studies report a significant association with an increased risk of OP and fractures (81, 82), others find no correlation or even contradictory outcomes (83, 84). These inconsistencies could stem from limitations inherent in observational studies, such as unaccounted or inaccurately measured confounding variables like gender, age, and menstrual status, leading to biases in the results. Bidirectional MR studies have suggested a causal relationship between chronic liver diseases and OP. However, further research is necessary to validate this causal link and better understand the complex interplay between chronic liver diseases and OP.

Our meta-analysis indicates a positive association between SHBG levels and the risk of OP, which is consistent with the conclusions drawn from the original analyses included in our study. Despite numerous proposed theories, the precise biological mechanisms linking SHBG to OP risk remain incompletely understood. SHBG, a glycoprotein, binds to sex hormones like testosterone and estradiol, thereby regulating their bioavailability (85). It is theorized that SHBG affects bone metabolism by altering the availability of free sex hormones, which are crucial for maintaining bone homeostasis. Testosterone promotes bone growth, whereas estradiol prevents bone loss. Elevated SHBG levels could potentially decrease sex hormone bioavailability, leading to increased bone turnover and lower BMD (86, 87). Additionally, SHBG might directly impact bone cells, as it has been observed to interact with receptors on osteoblasts and osteoclasts, possibly affecting their functions (88). In essence, it is possible that SHBG contributes to the risk of OP through its direct effects on bone cells and related factors in bone health.

### 4.2 Protective factors

BMR serves as a gauge of the body’s overall metabolism, playing a crucial role in sustaining normal physiological functions (33). As we age, functional decline becomes inevitable, affecting various bodily processes, including bone health. BMR could potentially be a modifiable element in decreasing the prevalence of OP. Evidence from a cross-sectional study indicates that a lower BMR correlates with a heightened risk of osteosarcopenia among postmenopausal women (89). Furthermore, another study posits that BMR, along with body fat, may serve as significant predictors for BMD at the femoral neck and spine in women aged over 50 (90). Two-sample MR analysis suggest that higher BMR may reduce the risk of OP (33). The increase in BMR may affect body composition by increasing energy expenditure, especially by increasing lean body mass (LBM), which is an important influencing factor of BMD. The increased LBM may have a positive impact on bones through mechanical loading, promoting bone formation and enhancing BMD (91). In addition, as age increases, BMR decreases, leading to a decrease in energy expenditure, which may result in changes in body composition such as a decrease in muscle mass and an increase in fat mass, thereby affecting BMD (92).

The GM is a complex and diverse assembly of microorganisms inhabiting the human gastrointestinal tract, playing a pivotal role in both health and disease (93). Recently, there has been a surge in scientific research focusing on the relationship between GM and OP. Studies have demonstrated that GM can affect the balance of Treg/Th17 cells and associated cytokines via the immune system, influencing both the intestinal and systemic immune responses. This ultimately establishes a dynamic equilibrium between osteoblasts and osteoclasts, which is essential for maintaining normal bone mass (94). A study revealed that patients with OP exhibited significantly different GM compositions compared to healthy controls (95). The results from our meta-analysis as well as other studies included in the systematic review all provide strong evidence supporting the causal relationship between GM and OP.

### 4.3 Factors with no significant correlation

As one of the risk factors of OP, it is of great scientific significance to explore the causal relationship between obesity and OP from the genetic level. Previous studies have found that obesity is a protective factor for OP (96, 97). However, it has also been suggested that obesity may cause the differentiation of bone marrow mesenchymal stem cells into adipose cell lines, resulting in the increase of bone marrow adipose tissue and the decrease of osteoblasts, while excess bone marrow adipose tissue may change the bone microenvironment and microstructure through the replacement of bone cells by adipose cells, resulting in the decrease of bone density (98). On the other hand, excessive adipose tissue in the bone marrow releases many pro-inflammatory molecules, many of which activate the RANK pathway to upregulate the formation and activation of osteoclasts (99).

The prevalence of sedentary behaviors is widespread across various industries and occupations, encompassing activities such as sitting, lying down, or engaging in a series of sedentary activities during awake time (100). While some observational studies suggest a link between increased sedentary time and a higher risk of reduced bone mass, the evidence is not uniform (101). The inconsistent results of observational studies may be due to confounding variables. For example, it has been demonstrated that increased TV-watching time is correlated with higher consumption of high-fat, high-sugar, and high-energy foods which are known to adversely affect bone health (102). Our meta-analysis did not establish a direct causal link between leisure sedentary behaviors and OP.

Diabetes mellitus, a prevalent endocrine disorder, is experiencing an increasing incidence trend. Hyperglycemia, a hallmark of diabetes, is implicated in numerous chronic complications, with OP being a common consequence that often leads to joint issues, persistent discomfort, and a heightened risk of disability (103). T2DM is known for its association with either normal or increased BMD. Paradoxically, it is also linked to reduced bone turnover and a greater fracture risk (104–106). Observational studies have not reached a consensus on T2DM’s impact on BMD; some report higher BMD in T2DM patients compared to non-diabetics (107, 108), while others note no significant relationship or even the contrary (109, 110). These discrepancies could stem from variations in study designs, medication effects, and confounding variables such as BMI. MR studies suggest that T2DM may lower OP incidence; however, this finding was not corroborated by our meta-analysis. Given the limitations of MR studies and T2DM’s negligible protective effect against OP, extensive RCTs are needed to clarify the potential causal link between T2DM and OP.

Metformin is a first-line drug for the treatment of type 2 diabetes, which is favored in the clinical environment because of its affordability, effectiveness and minimal side effects (111). There is growing evidence suggesting that, apart from its hypoglycemic properties, metformin also has a positive impact on OP (112). Various mechanisms have been postulated to elucidate metformin’s effect on bone health. At the cellular level, metformin fosters osteoblast differentiation by activating the AMP-activated protein kinase (AMPK) pathway, promoting the expression of Small Heterodimer Partner (SHP) and Runt-related transcription factor 2 (Runx2), and augmenting osteocalcin gene transcription (113). Concurrently, it deters osteoclast differentiation by enhancing osteoprotegerin (OPG) synthesis and curtailing receptor activator of nuclear factor-kappa B ligand (RANKL) production in osteoblasts (114). Nevertheless, a study has reported the absence of bone-forming effects of metformin in ovariectomized mice, casting doubts on its efficacy (115). The link between metformin usage and OP incidence is still under debate, necessitating further investigation to solidify this association.

SLE is a chronic autoimmune disorder marked by serum autoantibodies, leading to damage in multiple organs and tissues throughout the body (116). OP is one of its complications. The disease’s systemic inflammation is known to enhance bone resorption and reduce bone formation. Inflammatory responses mediated promote osteoclast differentiation and inhibit osteoblast activity, resulting in bone mass loss (117). Additionally, vitamin D deficiency, prevalent among SLE patients, impairs intestinal calcium absorption, further contributing to bone mass loss (118). Furthermore, glucocorticoids are widely used in treating SLE and its complications; their effects on bones are two-sided: long-term or massive use can promote OP development while also inhibiting bone destruction caused by systemic inflammatory response (119). A two-sample MR study indicates a lack of substantial evidence for a causal link between SLE and OP in European populations, suggesting that the association might be influenced by confounding factors. Conversely, genetic predisposition to SLE appears to have a positive causal link with OP in East Asian populations (23). Due to inconsistent conclusions from multiple studies, additional research is essential to clarify the causal dynamics between SLE and OP.

Iron is a crucial component for the human body, essential for mitochondrial function, DNA synthesis and repair, and cellular survival (120). Normally, the iron content in the human body remains relatively stable. However, abnormal fluctuations in iron levels can occur due to various factors, leading to disrupted iron metabolism which can impact the liver, heart, bones and joints. Research indicates that both excessive and insufficient levels of iron have negative effects on bone health (121, 122). Elevated iron levels can increase oxidative stress in the body, inhibit osteoblast formation, promote osteoclast differentiation, and ultimately reduce bone density (123). Some perspectives suggest that adverse effects of both iron deficiency and excess on osteoporosis risk are influenced by a U-shaped dose-dependent relationship between iron exposure and osteoblastogenesis as well as osteoblast activity (124). Despite the complexity of the relationship between OP and iron status, influenced by numerous factors, both MR findings and our meta-analysis indicate no genetic causation between OP and iron status (58).

### 4.4 Strengths and limitations

As far as we know, this systematic review and meta-analysis represents the first attempt to synthesize MR studies investigating the impact of various causal risk factors on OP risk. With the rapid increase of MR Studies, it is necessary and meaningful to conduct a summary analysis of heterogeneous studies with the same results.

This study possesses several strengths. The MR approach, as opposed to traditional epidemiological research, allows for causal inference at the genetic level, thereby significantly reducing biases stemming from confounding factors and reverse causation, ultimately enhancing result reliability. As MR is based on GWAS, the robustness of evidence is directly related to GWAS sample size. Our meta-analysis combined data from different GWAS focusing on exposure and outcome, expanding both participant base and sample size. Adherence to STROBE-MR guidelines enabled a comprehensive assessment of study quality. The findings indicated high-quality literature inclusion, ranging from study design to result discussion, with low risk of bias. In conducting our meta-analysis, we employed either fixed effects model or random effects model based on heterogeneity within included references. This approach ensures data robustness and underpins the reliability of our study’s results.

Nevertheless, our meta-analysis is not without limitations. The scarcity of MR studies on OP precluded the assessment of publication bias through funnel plot asymmetry and the performance of subgroup analyses by age, sex, and region. Secondly, significant heterogeneity among included studies necessitated cautious interpretation of results attributable to variations in study methodologies participants and locations. Finally, although the MR method provides evidence for the correlation between OP and related risk factors, this method itself also has some limitations. For example, linkage disequilibrium and pleiotropy may allow instrumental variables to affect results through multiple pathways, violating the MR’s assumption of “exclusion restriction assumption” and introducing bias. So the research results still need clinical studies and experimental verification.

### 4.5 Clinical implications

Considering the potential impact of RA, IBD, SHBG, asthma, depression, NAFLD, and PBC on OP risk, we suggest promoting increased physical activity, establishing regular lifestyle habits, and enhancing disease prevention awareness. Similarly, in light of the potential protective effects of BMR and GM against OP risk, we recommend prioritize strength training, ensure adequate protein intake and quality sleep for improved BMR, as well as maintaining a diverse diet and considering probiotic supplements to support a healthy gut microbiota balance.

### 4.6 Further studies

This review offers a comprehensive overview of current MR studies investigating risk factors for OP, while also highlighting several considerations for future research. Firstly, larger scale MR studies should be conducted in the future, prioritizing the use of the latest SNPs as instrumental variables to more accurately assess the impact of risk factors on OP development. In addition, in order to better understand the characteristics of OP risk factors, future research must cover different populations exposed to different environmental factors, not just the European cohort. Finally, although MR studies are powerful tools for epidemiological research, they may not necessarily represent the true causal effects. In the future, experimental validation is needed to consolidate causal relationships and utilize multi omics data such as genomics, transcriptomics, and metabolomics to further reveal the complex mechanisms underlying OP pathogenesis.

## 5 Conclusions

In conclusion, this study suggests that RA, IBD, CD, NAFLD, PBC, asthma, depression, and SHBG are identified as risk factors for OP, while BMR and GM are considered protective factors. The associations of obesity, SLE, metformin use, leisure sedentary behaviors, UC, serum iron levels, and T2DM with OP were found to be not statistically significant. Despite limitations such as limited population representativeness and heterogeneity of genetic tools, this study still has significant clinical implications. These findings offer valuable insights for implementing tertiary prevention in clinical practice and provide a potential direction for further research on OP.

## Data Availability

The original contributions presented in the study are included in the article/**Supplementary Material**. Further inquiries can be directed to the corresponding authors.
