# Supplementary material for: Mendelian randomization studies of risk and protective factors for osteoporosis: a systematic review and meta-analysis
Source: Front Endocrinol (Lausanne). 2025 Jan 16;15:1486188. doi: 10.3389/fendo.2024.1486188 (PMC11779621; doi:10.3389/fendo.2024.1486188)
Supplement: Supplementary file 1 [file DataSheet1.pdf]

Supplementary Material

Table S1. Search strategy used in each database searched.

**Database**	**Search strategy**	**Articles retrieved**
PubMed	(("Mendelian Randomization Analysis"[Mesh]) OR (Analysis, Mendelian Randomization[Title/Abstract]) OR (Mendelian Randomization[Title/Abstract])) AND (("osteoporosis"[Mesh]) OR (osteoporosis[Title/Abstract])) OR (osteoporoses[Title/Abstract]) OR (age-related bone loss[Title/Abstract]))	122
Web of science	#1.((TS=(Mendelian Randomization Analysis)) OR TS=(Analysis, Mendelian Randomization)) OR TS=(Mendelian Randomization))<br>#2.((TS=(osteoporosis) OR TS=(osteoporoses)) OR TS=(age-related bone loss))<br>#3. #2 AND #1	281
Embase	#1. 'osteoporosis'/exp<br>#2. osteoporosis:ab,ti OR osteoporoses:ab,ti OR 'age-related bone loss':ab,ti<br>#3. 'mendelian randomization analysis'/exp<br>#4. 'mendelian randomization analysis':ab,ti OR 'analysis, mendelian randomization':ab,ti OR 'mendelian randomization':ab,ti<br>#5. #1 OR #2<br>#6. #3 OR #4<br>#7. #5 AND #6	303

Table S2. Quality Assessment tool conducted based on adherence to the Strengthening the Reporting of Mendelian Randomization Studies (STROBE-MR) Guidelines for all 30 studies included in the meta-analysis. Each item is scored between 0 and 1 for each criterion to yield a total score. Upon conversion of the quality assessment score to a percentage, scores of < 75%, 75 to 85%, and > 85% were considered to indicate high, medium, and low risk of bias, respectively

**Study and year of publication**	1. Title& abstract	2. Background & objective	3. Design &data sources	4. Study sample	5. Selection of genetic variants	6. Primary analysis	7. Sensitivity analyses	8. Software and preregistration	9. Data presentation	10. Limitations, interpretation	Total score (out of 10)	% score
Zhao et al,2023 (1)	1	1	1	1	1	1	1	1	1	1	10	100
Wu et al,2024 (2)	1	1	1	1	1	1	1	1	1	1	10	100
Liu et al,2024 (3)	1	1	1	1	1	1	1	1	1	1	10	100
Chen et al,2024 (4)	1	1	1	1	1	1	1	1	1	1	10	100
Xu et al,2022 (5)	1	1	1	1	1	1	1	1	1	1	10	100
Cheng et al,2019 (6)	1	1	1	1	1	1	1	1	1	1	10	100
Tang et al,2023 (7)	1	1	1	1	1	1	1	1	1	1	10	100
Guo et al,2024 (8)	1	1	1	1	1	1	1	1	1	1	10	100
Wei et al,2023 (9)	1	1	1	1	1	1	1	1	1	1	10	100
Cai et al,2024 (10)	1	1	1	1	1	1	1	1	1	1	10	100
Zeng et al,2024 (11)	1	1	1	1	1	1	1	1	1	1	10	100
Chen et al,2023 (12)	1	1	1	1	1	1	1	1	1	1	10	100
Gagnon et al,2023 (13)	1	1	1	0.5	1	0.5	0.5	1	0.5	1	8	80
Martin et al,2022 (14)	1	1	1	1	0.5	1	0.5	1	1	1	9	90
Xu et al,2023 (15)	1	1	1	1	1	1	1	1	1	1	10	100

Dai et al,2023 (16)	1	1	1	1	1	1	1	1	1	1	10	100
Zhou et al,2023 (17)	1	1	1	1	1	1	1	1	1	1	10	100
Cui et al,2023 (18)	1	1	1	1	1	1	1	1	1	1	10	100
Zhou et al,2023 (19)	1	1	1	1	1	1	1	1	1	1	10	100
Cheng et al,2023 (20)	1	1	1	1	1	1	1	1	1	1	10	100
Huang et al,2023 (21)	1	1	1	1	1	1	1	1	1	1	10	100
Wu et al,2024 (22)	1	1	1	1	1	1	1	1	1	1	10	100
Deng et al,2023 (23)	1	1	1	1	1	1	1	1	1	1	10	100
Kasher et al,2022 (24)	0.5	1	1	1	1	1	1	1	0.5	1	9	90
Yu et al,2021 (25)	1	1	0.5	1	0.5	1	0.5	1	0.5	1	8	80
Sun et al,2024 (26)	1	1	1	1	1	1	1	1	1	1	10	100
Huang et al,2024 (27)	1	1	1	1	1	1	1	1	1	1	10	100
Shi et al,2024 (28)	1	1	1	1	1	1	1	1	0.5	1	9.5	95
Chen et al,2024 (29)	1	1	1	0.5	1	1	1	1	1	1	9.5	95
Ding et al,2024 (30)	1	1	1	0.5	1	1	1	1	1	1	9.5	95

Table S3. Mendelian randomization studies included in the meta-analyses of genetically predicted risk and protective factors in relation to osteoporosis.

Phenotype	Consortium (X)	Cases, n	SNPs,n	OR	LB	UB	p-value	p-value for MR-Egger intercept	p-value for heterogeneity analysis	Study, yr
Rheumatoid arthritis	GWAS-meta	56284	108	1.123	1.077	1.171	4.02×10^{-8}	0.571	0.388	Wu et al,2024 (22)
	BBJ	212453	11	1.10	1.06	1.14	＜0.001	0.737	0.526	Deng et al,2023 (23)
	UKB	5422	43	1.001	1.001	1.001	＜0.001	N/A	0.756	Kasher et al,2022 (24)
	BBJ	22514	N/A	1.130	1.050	1.216	1.04E-03	N/A	N/A	Yu et al,2021 (25)
**Rheumatoid arthritis**	Meta-analysis	296673		1.08	1.01	1.17	0.04			
Type 2 diabetes mellitus	BBJ	191764	63	0.92	0.86	0.99	0.016	0.729	0.369	Huang et al,2023 (21)
	FinnGen	212351	38	0.998	0.997	0.999	0.0056	0.299	0.08	Cheng et al,2023 (20)
**Type 2 diabetes mellitus**	Meta-analysis	700788		0.9676	0.8975	1.0430	0.39			
Inflammatory bowel disease	IIBDGC	6543	10	1.040	0.988	1.095	0.135	＞0.05	1.94E-01	Xu et al,2023 (15)
	IEU	34652	62	1.050	0.999	1.103	0.055	0.948	0.403	Dai et al,2023 (16)
	IIBDGC	59957	102	1.063	1.019	1.109	0.005	0.171	0.477	Dai et al,2023 (16)
**Inflammatory bowel disease**	Meta-analysis	101152		1.0525	1.0242	1.0817	0.0002			
Ulcerative colitis	IIBDGC	4853	7	0.992	0.932	1.056	0.801	＞0.05	2.91E-02	Xu et al,2023 (15)
	IEU	27432	31	1.021	0.963	1.083	0.484	0.422	0.562	Dai et al,2023 (16)
	IIBDGC	45975	48	1.065	0.975	1.099	0.257	0.076	0.341	Dai et al,2023 (16)
**Ulcerative colitis**	Meta-analysis	78260		1.018	0.9797	1.0579	0.36			

Crohn's disease	IIBDGC	5409	14	1.038	1.006	1.072	0.019	＞0.05	1.21E-01	Xu et al,2023 (15)
	IEU	20883	51	1.06	1.016	1.106	0.007	0.833	0.487	Dai et al,2023 (16)
	IIBDGC	40266	80	1.044	1.002	1.088	0.039	0.178	0.69	Dai et al,2023 (16)
**Crohn's disease**	Meta-analysis	66558		1.0453	1.0231	1.068	＜0.0001			
Non-alcoholic fatty liver disease	IEU	443204	9	0.984	N/A	N/A	0.834	0.21	0.508	Liu et al,2024 (3)
	GWAS-meta	397799	5	1.0021	1.0006	1.0037	0.007	0.346	0.831	Cui et al,2023 (18)
	UKB	32859	14	1.003	1.001	1.005	0.004	0.749	0.779	Zhou et al,2023 (19)
	The Million Veteran Program	218595	50	1.001	1	1.003	0.02	0.39	0.009	Zhou et al,2023 (19)
	FinnGen	64182	18	1.001	1	1.002	0.008	0.37	0.021	Zhou et al,2023 (19)
**Non-alcoholic fatty liver disease**	Meta-analysis	1156639		1.0014	1.0008	1.0020	＜0.0001			
Sex hormone binding globulin	GWAS-meta	368929	330	1.38	1.09	1.75	6.80E-03	0.94	2.39E-03	Sun et al,2024 (26)
	IEU	189473	208	1.479	1.144	1.912	0.003	N/A	0.252	Huang et al,2024 (27)
**Sex hormone binding globulin**	Meta-analysis	558402		1.4245	1.1973	1.6948	＜0.0001			
Basal metabolic rate	IEU	454874	439	0.9923	0.9898	0.9949	4.01E-09	0.0929	4.95E-07	Zhou et al,2023 (17)
	IEU	454874	462	0.9939	0.9911	0.9966	1.04E-05	0.2339	2.38E-03	Zhou et al,2023 (17)
**Basal metabolic rate**	Meta-analysis	454874		0.9931	0.9912	0.9949	＜0.0001			
Body mass index	IEU	339224	N/A	0.85	0.62	1.17	0.324	N/A	0.702	Martin et al,2022 (14)
	IEU	339224	N/A	1.17	1.1	1.24	3.00E-06	N/A	1.00E-93	Martin et al,2022 (14)
	IEU	339224	N/A	0.73	0.63	0.84	6.00E-05	N/A	N/A	Martin et al,2022 (14)
**Body mass index**	Meta-analysis	339224		0.91	0.63	1.30	0.59			
Body fat percentage	UKB	442278	N/A	1.02	0.82	1.26	0.893	N/A	0.399	Martin et al,2022 (14)
	UKB	442278	N/A	1.16	1.11	1.21	5.00E-11	N/A	0.00E+00	Martin et al,2022 (14)

	UKB	442278	N/A	0.8	0.72	0.89	3.00E-05	N/A	N/A	Martin et al,2022 (14)
**Body fat percentage**	Meta-analysis	442278		0.98	0.75	1.29	0.90			
Favourable adiposity	Martin et al, GWAS	442278	N/A	1.71	0.75	3.87	0.209	N/A	0.298	Martin et al,2022 (14)
	Martin et al, GWAS	442278	N/A	1.03	0.84	1.27	0.764	N/A	9.00E-102	Martin et al,2022 (14)
	Martin et al, GWAS	442278	N/A	1.55	0.92	2.61	0.105	N/A	N/A	Martin et al,2022 (14)
**Favourable adiposity**	Meta-analysis	442278		1.11	0.92	1.34	0.26			
Unfavourable adiposity	Martin et al, GWAS	442278	N/A	0.95	0.55	1.65	0.868	N/A	0.941	Martin et al,2022 (14)
	Martin et al, GWAS	442278	N/A	1.2	1.02	1.4	0.031	N/A	3.00E-124	Martin et al,2022 (14)
	Martin et al, GWAS	442278	N/A	0.7	0.52	0.95	0.027	N/A	N/A	Martin et al,2022 (14)
**Unfavourable adiposity**	Meta-analysis	442278		0.94	0.63	1.39	0.75			
Primary biliary cholangitis	Cordell et al, GWAS	24510	32	1.049	1.017	1.082	0.002	0.103	0.61	Zhao et al,2023 (1)
	Cordell et al, GWAS	31228	23	1.096	1.052	1.142	0.001	0.779	0.221	Liu et al,2024 (3)
	GWAS-meta	24510	37	1.04	1.016	1.064	0.001	0.061	0.6	Wu et al,2024 (2)
	GWAS-meta	6778	13	1.059	1.023	1.096	0.001	0.125	0.478	Wu et al,2024 (2)
**Primary biliary cholangitis**	Meta-analysis	62516		1.05	1.04	1.07	＜0.0001			
Serum iron	GWAS-meta	163511	35	0.99	0.85	1.15	0.89	0.137	0.393	Xu et al,2022 (5)
	NHGRI-EBI GWAS	48972	9	0.98	0.81	1.19	0.815	0.68	0.06	Cheng et al,2019 (6)
**Serum iron**	Meta-analysis	212483		0.99	0.88	1.11	0.82			
Depression	UKB	322580	14	2.121	0.28	16.084	0.476	0.421	0.383	Tang et al,2023 (7)
	PGC	500199	47	1.0008	1.0004	1.0072	0.013	N/A	N/A	Guo et al,2024 (8)
	PGC	500199	11	1.0022	1.0005	1.004	0.0136	0.3411	N/A	Guo et al,2024 (8)
**Depression**	Meta-analysis	822779		1.0009	1.0005	1.0012	＜0.0001			
Metformin	UKB	462933	43	0.018	0.0023	0.144	0.000153	8.19E-01	4.69E-01	Wei et al,2023 (9)
	UKB	456276	34	0.859	0.774	0.953	0.004	0.089	0.146	Cai et al,2024 (10)

**Metformin**	Meta-analysis	919209		0.1433	0.0033	6.2665	0.31			
Leisure sedentary behaviors	van de Vegte et al,GWAS	437887	27	1.004	0.996	1.011	0.303	0.293	0.572	Chen et al,2024 (4)
	van de Vegte et al,GWAS	360895	12	1.005	0.992	1.017	0.461	0.09	0.247	Chen et al,2024 (4)
	van de Vegte et al,GWAS	310555	9	0.995	0.977	1.012	0.551	0.054	0.139	Chen et al,2024 (4)
**Leisure sedentary behaviors**	Meta-analysis	1109337		1.0031	0.9968	1.0096	0.34			
Gut microbiota (NB1n)	MiBioGen	18340	3	0.999	N/A	N/A	0.555	N/A	N/A	Gagnon et al,2023 (13)
	MiBioGen	18340	15	0.998	0.996	1	0.0154	0.46	0.554	Zeng et al,2024 (11)
	MiBioGen	18340	14	0.998	0.997	0.999	0.038	0.413	0.987	Chen et al,2023 (12)
**Gut microbiota (NB1n)**	Meta-analysis	18340		0.9981	0.9972	0.9989	＜0.0001			
Asthma	UKB	N/A	53	1.007	1.003	1.013	0.039	0.482	0.323	Ding et al,2024 (30)
	UKB	N/A	30	1.012	1.002	1.023	0.018	0.384	0.584	Ding et al,2024 (30)
	GWAS Catalog	N/A	13	1.169	1.002	1.362	0.047	0.474	0.162	Chen et al,2024 (29)
**Asthma**	Meta-analysis	N/A		1.0104	1.0005	1.0203	0.04			
Systemic lupus erythematosus	Wang YF et al,GWAS	12653	31	1.04	1.01	1.07	0.01	0.627	0.847	Shi et al,2024 (28)
	Bentham J et al,GWAS	14267	31	1	1	1	0.272	0.148	0.004	Shi et al,2024 (28)
**Systemic lupus erythematosus**	Meta-analysis	26920		1.02	0.98	1.06	0.35			

UKB, UK Biobank; BBJ, BioBank Japan; GWAS, genome-wide association studies; IEU, IEU OpenGWAS project; IIBDGC, International Inflammatory Bowel Disease Genetics Consortium; NHGRI-EBI, National Human Genome Research Institute and European Bioinformatics Institute; PGC, Psychiatric Genomics Consortium; MiBioGen, Microbial Biogeography and Genomics; LB, low bound; MR, Mendelian randomization; N/A, not available; OR, odds ratio; SNP, single nucleotide polymorphism; UB, up bound.

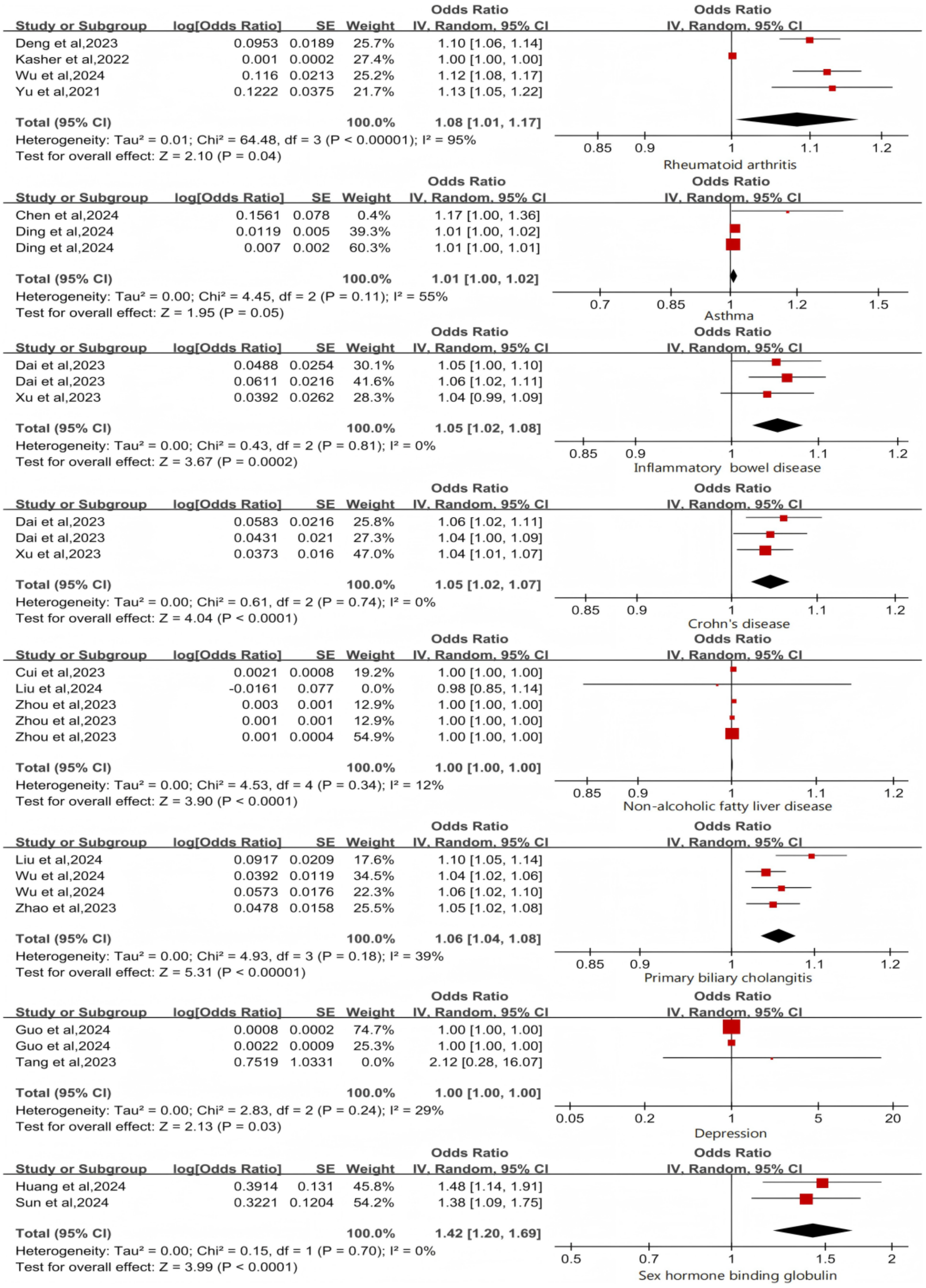

Figure S1 Forest plot of random effects model for risk factors of osteoporosis.

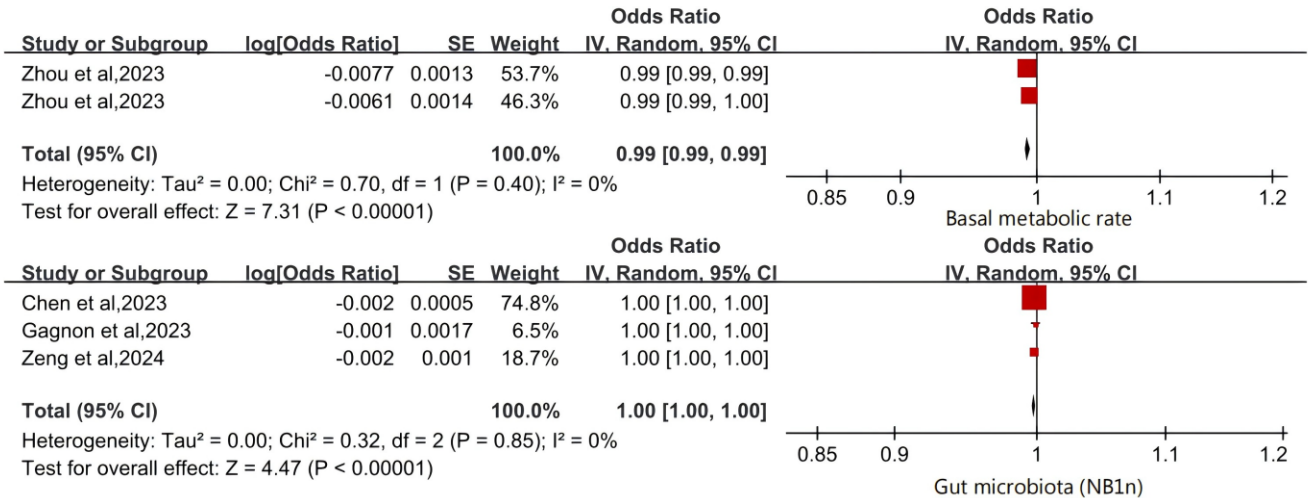

Figure S2 Forest plot of random effects model for protective factors of osteoporosis.

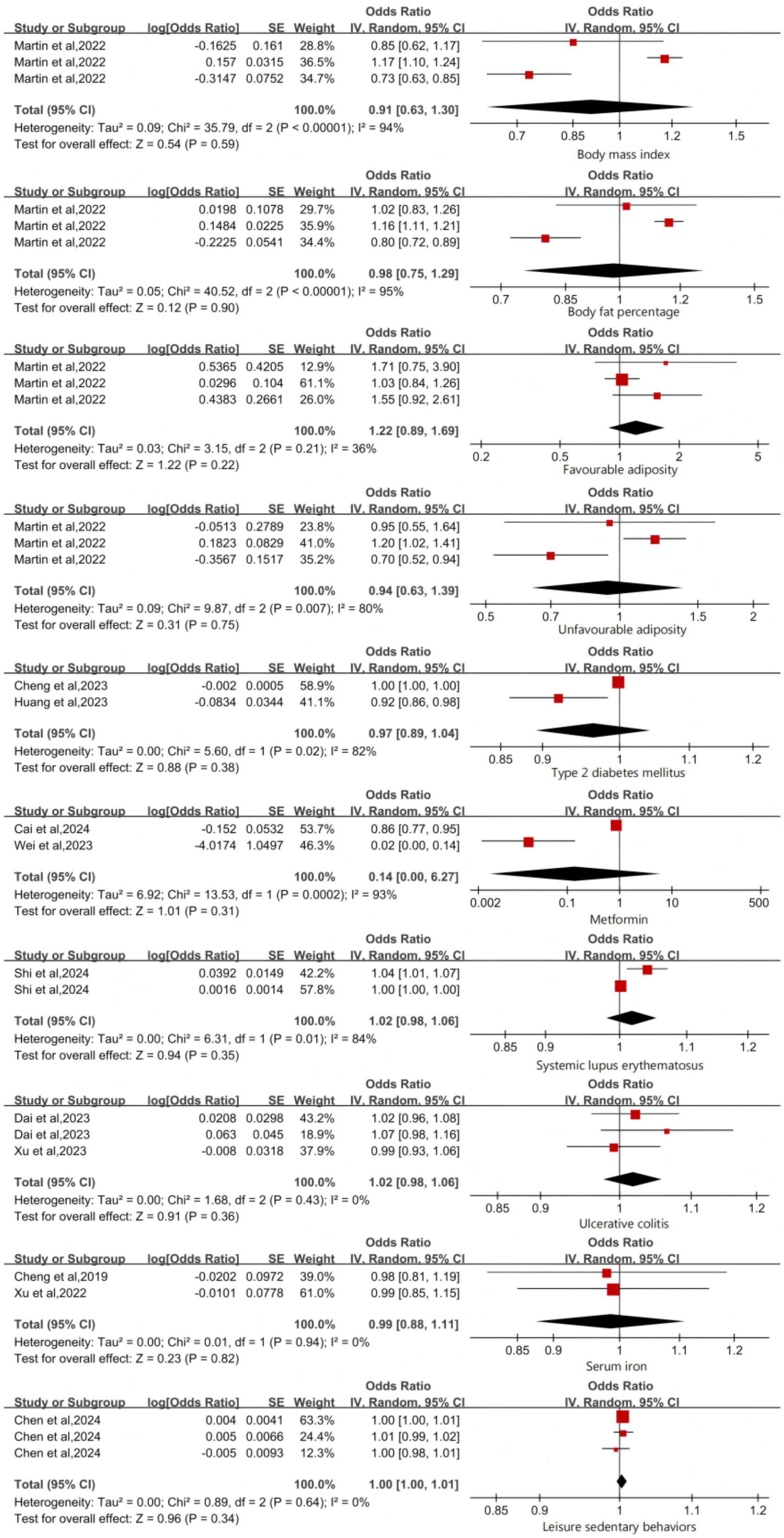

Figure S3 Forest plot of a random effects model of factors with no significant association with osteoporosis.

References

1. Zhao D, Li G, Bai W, Teng J, Yan B, Han C. Primary biliary cirrhosis and osteoporosis: a bidirectional two-sample Mendelian randomization study. *Frontiers in immunology*. (2023) 14:1269069. Epub 2024/01/02. doi: 10.3389/fimmu.2023.1269069.

2. Wu Y, Qian Q, Liu Q, Wang R, Pu X, Li Y, et al. Osteoporosis and Primary Biliary Cholangitis: A Trans-ethnic Mendelian Randomization Analysis. *Clinical reviews in allergy & immunology*. (2024). Epub 2024/03/30 20:53. doi: 10.1007/s12016-024-08986-4.

3. Liu Y, Yuan M, He J, Cai L, Leng A. The Impact of Non-alcohol Fatty Liver Disease on Bone Mineral Density is Mediated by Sclerostin by Mendelian Randomization Study. *Calcified tissue international*. (2024) 114(5):502-12. Epub 2024/03/31. doi: 10.1007/s00223-024-01204-5.

4. Chen Y, Yu J, Li W, Wang L, Zhou X, Zhuang C, et al. Potential causal association between leisure sedentary behaviors and osteoporosis: A two-sample Mendelian randomization analysis. *Medicine*. (2024) 103(12):e37467. Epub 2024/03/22. doi: 10.1097/md.0000000000037467.

5. Xu J, Ma J, Chen J, Zhang S, Zheng C, Si H, et al. No genetic causal association between iron status and osteoporosis: A two-sample Mendelian randomization. *Frontiers in endocrinology*. (2022) 13:996244. Epub 2022/12/27. doi: 10.3389/fendo.2022.996244.

6. Cheng WW, Zhu Q, Zhang HY. Mineral Nutrition and the Risk of Chronic Diseases: A Mendelian Randomization Study. *Nutrients*. (2019) 11(2). Epub 2019/02/15. doi: 10.3390/nu11020378.

7. Tang F, Wang S, Zhao H, Xia D, Dong X. Mendelian randomization analysis does not reveal a causal influence of mental diseases on osteoporosis. *Frontiers in endocrinology*. (2023) 14:1125427. Epub 2023/05/08. doi: 10.3389/fendo.2023.1125427.

8. Guo X, She Y, Liu Q, Qin J, Wang L, Xu A, et al. Osteoporosis and depression in perimenopausal women: From clinical association to genetic causality. *Journal of affective disorders*. (2024) 356:371-8. Epub 2024/04/13. doi: 10.1016/j.jad.2024.04.019.

9. Wei YK, Chen PB, Ju LL, Deng GH. Causal association of metformin and osteoporosis: A 2-sample Mendelian randomization study. *Medicine*. (2023) 102(43):e35191. Epub 2023/10/31. doi: 10.1097/md.0000000000035191.

10. Cai Y, Jun G, Zhuang X. Metformin treatment reduces the incidence of osteoporosis: a two-sample Mendelian randomized study. *Osteoporosis international : a journal established as result of cooperation between the European Foundation for Osteoporosis and the National Osteoporosis Foundation of the USA*. (2024). Epub 2024/03/27. doi: 10.1007/s00198-023-07013-0.

11. Zeng HQ, Li G, Zhou KX, Li AD, Liu W, Zhang Y. Causal link between gut microbiota and osteoporosis analyzed via Mendelian randomization. *European review for medical and pharmacological sciences*. (2024) 28(2):542-55. Epub 2024/02/02. doi: 10.26355/eurrev_202401_35052.

12. Chen S, Han H, Sun X, Zhou G, Zhou Q, Li Z. Causal effects of specific gut microbiota on musculoskeletal diseases: a bidirectional two-sample Mendelian randomization study. *Frontiers in microbiology*. (2023) 14:1238800. Epub 2023/09/04. doi: 10.3389/fmicb.2023.1238800.

13. Gagnon E, Mitchell PL, Manikpurage HD, Abner E, Taba N, Esko T, et al. Impact of the gut microbiota and associated metabolites on cardiometabolic traits, chronic diseases and human longevity: a Mendelian randomization study. *Journal of translational medicine*. (2023) 21(1):60. Epub 2023/02/01. doi: 10.1186/s12967-022-03799-5.

14. Martin S, Tyrrell J, Thomas EL, Bown MJ, Wood AR, Beaumont RN, et al. Disease consequences of higher adiposity uncoupled from its adverse metabolic effects using Mendelian randomisation. *eLife*. (2022) 11. Epub 2022/01/26. doi: 10.7554/eLife.72452.

15. Xu D, Chen Y, Gao X, Xie W, Wang Y, Shen J, et al. The genetically predicted causal relationship of inflammatory bowel disease with bone mineral density and osteoporosis: evidence from two-sample Mendelian randomization. *Frontiers in immunology*. (2023) 14:1148107. Epub 2023/06/05.

doi: 10.3389/fimmu.2023.1148107.
16. Dai Z, Xu W, Ding R, Peng X, Shen X, Song J, et al. Two-sample Mendelian randomization analysis evaluates causal associations between inflammatory bowel disease and osteoporosis. *Frontiers in public health*. (2023) 11:1151837. Epub 2023/06/12. doi: 10.3389/fpubh.2023.1151837.
17. Zhou J, Ye Z, Wei P, Yi F, Ouyang M, Xiong S, et al. Effect of basal metabolic rate on osteoporosis: A Mendelian randomization study. *Frontiers in public health*. (2023) 11:1096519. Epub 2023/02/24. doi: 10.3389/fpubh.2023.1096519.
18. Cui A, Xiao P, Fan Z, Lei J, Han S, Zhang D, et al. Causal association of NAFLD with osteoporosis, fracture and falling risk: a bidirectional Mendelian randomization study. *Frontiers in endocrinology*. (2023) 14:1215790. Epub 2023/08/25. doi: 10.3389/fendo.2023.1215790.
19. Zhou Y, Ni Y, Wang Z, Prud'homme GJ, Wang Q. Causal effects of non-alcoholic fatty liver disease on osteoporosis: a Mendelian randomization study. *Frontiers in endocrinology*. (2023) 14:1283739. Epub 2023/12/27. doi: 10.3389/fendo.2023.1283739.
20. Cheng L, Wang S, Tang H. Type 2 diabetes mellitus plays a protective role against osteoporosis --mendelian randomization analysis. *BMC musculoskeletal disorders*. (2023) 24(1):444. Epub 2023/06/03. doi: 10.1186/s12891-023-06528-1.
21. Huang G, Chen X, Chen Y, Liu W, Chen C, Song W, et al. Causal relationship between type 2 diabetes mellitus and bone mineral density: a Mendelian randomization study in an East Asian population. *Osteoporosis international : a journal established as result of cooperation between the European Foundation for Osteoporosis and the National Osteoporosis Foundation of the USA*. (2023) 34(10):1719-27. Epub 2023/06/12. doi: 10.1007/s00198-023-06807-6.
22. Wu RQ, Zhou Y, Xia T, Zhang C, Yang QP, Zhang X, et al. Mendelian randomization study on the associatoion between rheumatoid arthritis and osteoporosis and bone mineral density. *Chinese Journal of Tissue Engineering Research*. (2024) 28(23):3715-21. doi: 10.12307/2024.412.
23. Deng Y, Wong MCS. Association Between Rheumatoid Arthritis and Osteoporosis in Japanese Populations: A Mendelian Randomization Study. *Arthritis & rheumatology (Hoboken, NJ)*. (2023) 75(8):1334-43. Epub 2023/04/12. doi: 10.1002/art.42502.
24. Kasher M, Williams FMK, Freidin MB, Malkin I, Cherny SS, Livshits G. Understanding the complex genetic architecture connecting rheumatoid arthritis, osteoporosis and inflammation: discovering causal pathways. *Human molecular genetics*. (2022) 31(16):2810-9. Epub 2022/03/30. doi: 10.1093/hmg/ddac061.
25. Yu XH, Yang YQ, Cao RR, Cai MK, Zhang L, Deng FY, et al. Rheumatoid arthritis and osteoporosis: shared genetic effect, pleiotropy and causality. *Human molecular genetics*. (2021) 30(21):1932-40. Epub 2021/06/17. doi: 10.1093/hmg/ddab158.
26. Sun K, Li M, Wu Y, Wu Y, Zeng Y, Zhou S, et al. Exploring Causal Relationships between Leukocyte Telomere Length, Sex Hormone-Binding Globulin Levels, and Osteoporosis Using Univariable and Multivariable Mendelian Randomization. *Orthopaedic surgery*. (2024) 16(2):320-8. Epub 2023/12/12. doi: 10.1111/os.13947.
27. Huang W, Xiao Y, Zhang L, Liu H. The Association Between SHBG and Osteoporosis: A NHANES Cross-Sectional Study and A Bidirectional Mendelian Randomization. *Calcified tissue international*. (2024) 114(3):237-45. Epub 2023/12/05. doi: 10.1007/s00223-023-01166-0.
28. Shi YK, Yuan KH, Fu ZM, Hu RR, Wang H. The Relationship Between Systemic Lupus Erythematosus and Osteoporosis Based on Different Ethnic Groups: a Two-Sample Mendelian Randomization Analysis. *Calcified tissue international*. (2024) 114(4):386-96. Epub 2024/02/15. doi: 10.1007/s00223-024-01190-8.
29. Chen L, Li C, Chen H, Xie Y, Su N, Luo F, et al. Cross-sectional studies of the causal link between asthma and osteoporosis: insights from Mendelian randomization and bioinformatics analysis.

Osteoporosis international : a journal established as result of cooperation between the European Foundation for Osteoporosis and the National Osteoporosis Foundation of the USA. (2024). Epub 2024/03/02. doi: 10.1007/s00198-024-07037-0.
30. Ding W, Huang Y, Li G, Dong Y, Li X, Wu M, et al. Higher risk of osteoporosis in adult-onset asthma than childhood-onset asthma: from genetic and prospective evidence. *Osteoporosis international : a journal established as result of cooperation between the European Foundation for Osteoporosis and the National Osteoporosis Foundation of the USA*. (2024) 35(4):659-68. Epub 2023/12/23. doi: 10.1007/s00198-023-07004-1.
